# Investigating the relationship between mitochondrial genetic variation and cardiovascular-related traits to develop a framework for mitochondrial phenome-wide association studies

**DOI:** 10.1186/1756-0381-7-6

**Published:** 2014-04-15

**Authors:** Sabrina L Mitchell, Jacob B Hall, Robert J Goodloe, Jonathan Boston, Eric Farber-Eger, Sarah A Pendergrass, William S Bush, Dana C Crawford

**Affiliations:** 1 Center for Human Genetics Research, Vanderbilt University Medical Center, Nashville, TN 37232, USA; 2 Department of Molecular Physiology and Biophysics, Vanderbilt University Medical Center, Nashville, TN 37232, USA; 3 Center for Systems Genomics, Department of Biochemistry and Molecular Biology, The Pennsylvania State University, University Park, PA 16802, USA; 4 Department of Biomedical Informatics, Vanderbilt University Medical Center, Nashville, TN 37232, USA

**Keywords:** Mitochondrial DNA variation, mtSNP, PheWAS, GCTA, Mixed modeling, Polygenic analysis

## Abstract

**Background:**

Mitochondria play a critical role in the cell and have DNA independent of the nuclear genome. There is much evidence that mitochondrial DNA (mtDNA) variation plays a role in human health and disease, however, this area of investigation has lagged behind research into the role of nuclear genetic variation on complex traits and phenotypic outcomes. Phenome-wide association studies (PheWAS) investigate the association between a wide range of traits and genetic variation. To date, this approach has not been used to investigate the relationship between mtDNA variants and phenotypic variation. Herein, we describe the development of a PheWAS framework for mtDNA variants (mt-PheWAS). Using the Metabochip custom genotyping array, nuclear and mitochondrial DNA variants were genotyped in 11,519 African Americans from the Vanderbilt University biorepository, BioVU. We employed both polygenic modeling and association testing with mitochondrial single nucleotide polymorphisms (mtSNPs) to explore the relationship between mtDNA variants and a group of eight cardiovascular-related traits obtained from de-identified electronic medical records within BioVU.

**Results:**

Using polygenic modeling we found evidence for an effect of mtDNA variation on total cholesterol and type 2 diabetes (T2D). After performing comprehensive mitochondrial single SNP associations, we identified an increased number of single mtSNP associations with total cholesterol and T2D compared to the other phenotypes examined, which did not have more significantly associated SNPs than would be expected by chance. Among the mtSNPs significantly associated with T2D we identified variant mt16189, an association previously reported only in Asian and European-descent populations.

**Conclusions:**

Our replication of previous findings and identification of novel associations from this initial study suggest that our mt-PheWAS approach is robust for investigating the relationship between mitochondrial genetic variation and a range of phenotypes, providing a framework for future mt-PheWAS.

## Background

As the primary energy producers of the cell, mitochondria are critical to cellular fitness. While energy production is the function most often associated with mitochondria, the organelle plays a vital role in other cellular processes including cholesterol synthesis, fatty acid oxidation, ammonia detoxification, calcium homeostasis, and apoptosis, suggesting that mitochondrial dysfunction could have far-reaching effects. Indeed, mitochondrial dysfunction has been observed in a variety of diseases, including amyotrophic lateral sclerosis (ALS)*,* Huntington’s disease, Alzheimer’s disease, and various types of cancer [1].

Mitochondria maintain their own DNA separate from the nuclear genome. The human mitochondrial genome consists of a double-stranded, circular chromosome spanning 16,569 base pairs. This maternally inherited, compact genome contains 37 genes encoding 2 ribosomal RNAs, 22 transfer RNAs, and 13 protein-encoding genes that are essential components of the oxidative phosphorylation complexes. Rare mutations in mitochondrial DNA (mtDNA) give rise to a spectrum of diseases, including Leber’s Hereditary Optic Neuropathy (LHON), Myoclonic Epilepsy with Ragged Red Fibers (MERRF), Maternally Inherited Diabetes and Deafness (MIDD), and prostate cancer [2]. Common mtDNA variation has also has been associated with common complex diseases, including type 2 diabetes (T2D) [3,4], multiple cancers [5], Alzheimer’s [6], and Parkinson’s [7]. Furthermore, mitochondrial single nucleotide polymorphisms (mtSNPs) have been associated with quantitative trait variance among phenotypes, such as triglycerides [8] and HDL-C [9], both known to play a role in cardiovascular disease. The range of phenotypes associated with mtDNA variation underscores the pleiotropic nature of mitochondrial genetic variation.

Phenome-Wide Association Studies (PheWAS) have been used to explore the association between nuclear single nucleotide polymorphisms (SNPs) and a wide array of phenotypes. To date, PheWAS have successfully used electronic-medical record (EMR) data and large population-based survey data [10-15] to replicate previously reported genome-wide association study (GWAS) findings, as well as to uncover intriguing novel associations. These studies have also identified potential pleiotropy, in which single SNPs are associated with multiple phenotypes [16]. However, thus far, the PheWAS approach has not been used to explore the relationship between mitochondrial SNPs (mtSNPs) and multiple phenotypes.

While there has been some research focused on identifying the impact of mtDNA variants on a number of complex traits, this relationship has been primarily explored through association testing with single traits or outcomes. Further work is needed to better characterize the relationship between mitochondrial genetic variation and a wide range of outcomes; this can be done by applying the PheWAS approach. Thus, the goal of this work was to develop a framework for implementing a mitochondrial PheWAS (mt-PheWAS). A method yet to be explored for characterizing the relationship between mitochondrial variation and phenotypes, in a PheWAS context, is the use of polygenic modeling [17], which can provide an estimate of heritability by considering the amount of phenotypic variation explained by genotypic variation. In this work, we demonstrate the utility of polygenic modeling for prioritizing phenotypic outcomes prior to pursuing mtSNP association analysis using a PheWAS approach. Within this framework, polygenic modeling, as well as haplogroup and mtSNP association tests can be used singly or in combination to explore the relationship between mtDNA variation and a range of phenotypes in the context of mt-PheWAS. Applying the PheWAS approach will allow for hypothesis-generation about the role of mtDNA variation in human health and disease.

To test our mt-PheWAS framework, we explored the relationship between mtDNA variation and eight cardiovascular-related phenotypes using Metabochip genotype data from African Americans in the Vanderbilt University biorepository, BioVU. We began with a polygenic approach, employing mixed linear modeling to investigate the overall contribution of mitochondrial genetic variation on trait variance. Single mtSNP association analysis was then performed with all eight traits to identify specific genotype-phenotype associations. Using the polygenic approach we identified two traits with evidence for association with mitochondrial genetic variation. Our single mtSNP association analysis confirmed the correlation between the number of significant mtSNPs observed for each trait and the overall proportion of trait variance explained by mitochondrial genetic variation, suggesting that our proposed mt-PheWAS framework, which includes a phenotype filtering step via polygenic modeling, is a valid approach for investigating the effect of mtDNA variation for a range of phenotypes.

## Methods

### Study population and phenotypes

The Vanderbilt University Medical Center (VUMC) biorepository, BioVU, contains more than 170,000 individual de-identified DNA samples linked to a de-identified version of the EMR, known as the synthetic derivative [18]. As part of the Population Architecture using Genomics and Epidemiology I (PAGE I) study, the Epidemiologic Architecture for Genes Linked to Environment (EAGLE) study has genotyped 15,863 BioVU samples (EAGLE BioVU) consisting of individuals 18 years and older from diverse populations, including 11,519 African Americans. All samples were genotyped on the Metabochip, a custom genotyping array containing 196,725 SNPs, the majority of which were chosen based on previous associations with metabolic, cardiovascular, and anthropometric traits, as well as to fine map the regions around these previously associated variants [19]. Among the variants on the Metabochip are 135 mtSNPs, including a putative T2D mtSNP and a rare MIDD mutation [19,20]. Genotyping was performed by the Center for Human Genetics DNA Resources Core at VUMC, and quality control was performed as previously described [21], resulting in 192,139 autosomal SNPs and 130 mtSNPs for use in this analysis.

In this pilot PheWAS, we accessed the African American samples in EAGLE BioVU and selected eight cardiovascular-related traits for analysis, including: body mass index (BMI), total cholesterol, high-density lipoprotein cholesterol (HDL-C), low-density lipoprotein cholesterol (LDL-C), triglycerides, mean corpuscular hemoglobin (MCH), T2D, and hypertension. BMI was defined by manually excluding extreme outliers that were likely a result of conversion measure inconsistencies within the EMR. Total cholesterol, HDL-C, LDL-C, and triglycerides were reported in milligrams per deciliter (mg/dL). Mean corpuscular hemoglobin was reported in picograms per cell (pg/cell). To obtain values for each quantitative trait, the median value for each year was determined and then the median of all years was stored as a single value per individual. For all continuous traits, only a single observation within the synthetic derivative was required for an individual to be included in the analysis. Cases and controls for T2D, were defined as previously described [22], except for the inclusion of those with a family history of diabetes as controls. Hypertension was dichotomized as case or control, in which cases were defined as having a systolic blood pressure greater than or equal to 140 mmHg, having a diastolic blood pressure greater than or equal to 90 mmHg, or currently taking any hypertension medication. Controls were selected from the remaining samples, excluding individuals with a history of prescribed hypertension medications. Age is reported as the median age, in years, for each individual. Study population characteristics are presented in Table 1.

**Table 1:** Characteristics of study population

**Trait/Phenotype**	**Sample size**	**Mean/%**	**SD**
Age (years)	9,559	46.1	16.8
Sex (% female)	9,559	65.4%	—
Body Mass Index (kg/m^2^)	7,965	28.8	6.6
Total Cholesterol (mg/dL)	5,075	179.2	38.9
HDL-C (mg/dL)	4,792	52.7	16.9
LDL-C (mg/dL)	4,731	102.1	35.3
Triglycerides (mg/dL)	4,924	116.6	69.3
Mean Corpuscular Hemoglobin (pg/cell)	9,559	28.4	2.6
Hypertension (Cases/Controls)	6,147/2,664	—	—
Type 2 Diabetes (Cases/Controls)	1,338/8,151	—	—

The sample size, mean, and standard deviation (SD) are shown for continuous traits. Sample size is the number of African American individuals in EAGLE BioVU with data available for a given trait. The numbers of cases and controls are shown for each binary trait.

### Polygenic modeling

We performed polygenic analysis using mixed-modeling to determine the overall contribution of mitochondrial genetic variation captured by the Metabochip to the variance of the eight selected traits. By first using polygenic modeling, phenotypes can be prioritized or filtered based on putative contribution of mtDNA variation, assuming that a lack of evidence for influence of mtDNA variation implies a reduced chance of identifying specific single mtSNP associations. Genome-wide Complex Trait Analysis (GCTA) [17], which employs a mixed linear model to estimate the overall impact of genetic variation on trait variance, was used for the polygenic analysis. A key component of GCTA is its use of a genetic relationship matrix (GRM) to estimate pair-wise relatedness using a collection of genotypes within a dataset, based on additive genetic effects. However, as opposed to the diploid nuclear genome, the mitochondrial genome is haploid, so mitochondrial GRMs are calculated based on allelic sharing, rather than additive effects. Unlike typical GWAS, in which the effects of single SNPs are calculated independently, GCTA evaluates the relative levels of genomic sharing between individuals, creating a GRM and associating this relatedness to quantitative trait variation or binary trait risk.

For each trait, GCTA was used to fit a mixed linear model and estimate the proportion of variance explained (PVE) by nuclear and mitochondrial genetic variation. Mixed models consist of fixed and random effects. Here, the fixed effects included age, sex, and the first two nuclear principal components (PCs) as covariates. Eigenstrat was used to generate nuclear PCs from the 192,139 autosomal SNPs that passed QC. The random effect is empirical genetic relatedness, which was estimated using GRMs, created via GCTA, for the nuclear and mitochondrial genomes, using 192,139 and 130 SNPs, respectively.

Expectation-maximization (EM), the iterative method for finding the maximum likelihood estimate, was used for restricted maximum likelihood (REML) analysis using GCTA. REML provides estimates of the proportion of trait variance explained by genetic variation in the nuclear and/or mitochondrial genome. A likelihood ratio test (LRT) was performed comparing full and reduced models to determine the significance of mitochondrial genetic variation on the overall mixed linear model for each trait. The likelihood ratio test measures the significance of a specific model component. The full model contained both a nuclear and mitochondrial GRM, while the reduced model contained only a nuclear GRM. Here, we used the likelihood ratio test to evaluate the contribution of mitochondrial genetic variation captured by the Metabochip to the phenotypic variation or risk observed in the selected cardiovascular-related traits.

### Single mtSNP analysis

We performed single mtSNP analysis for all traits to determine if filtering based on results from our polygenic analysis was beneficial. We excluded 44 mtSNPs with a minor allele frequency less than 1%, leaving 86 mtSNPs available for the single mtSNP association analysis. PLINK [23] was used to perform linear or logistic regression for continuous and binary traits, respectively, to investigate mtSNP-phenotype associations. Both linear and logistic regression models were adjusted for age, sex, and the first two nuclear PCs. Haplogroup association analysis was not carried out in this study. While the mtDNA coverage on the Metabochip can be used to infer haplogroups, it is more reliable for classifying populations of European descent than for other ancestries due to the percent mitochondrial genetic variation captured [19,20].

## Results

### Polygenic modeling

**Figure 1:**
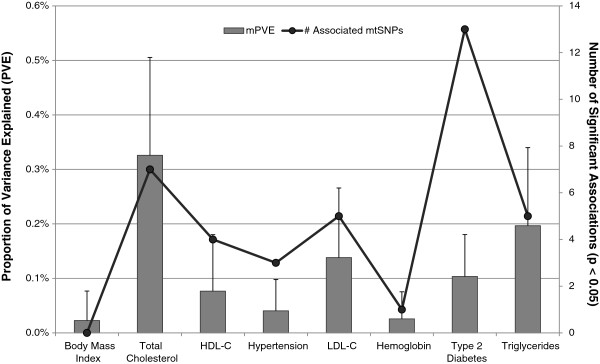
**Correlation of mitochondrial PVE and number of significantly associated mtSNPs.** The proportion of variance explained by mitochondrial genetic variation (mPVE), calculated using GCTA, is shown on the primary y-axis (bars shown represent standard error). The secondary y-axis displays the number of significantly associated SNPs observed for each trait, plotted as black dots.

All eight traits in our analysis had relatively low PVE by nuclear and mitochondrial variation. Nuclear PVE ranged from 0.33% for MCH to 2.95% for triglycerides. Mitochondrial PVE ranged from 0.02% for MCH to 0.33% for total cholesterol (Figure 1). The likelihood ratio tests performed to assess the relative significance of mitochondrial genetic variation yielded a significant p-value of 0.046 for total cholesterol. Additionally, T2D had a suggestive p-value of 0.055. The likelihood ratio test p-value for other traits ranged from 0.15 to 0.50 (Additional file 1), suggesting that mitochondrial variation captured by the Metabochip does not contribute significantly to those traits.

### Single mtSNP tests of association

**Figure 2:**
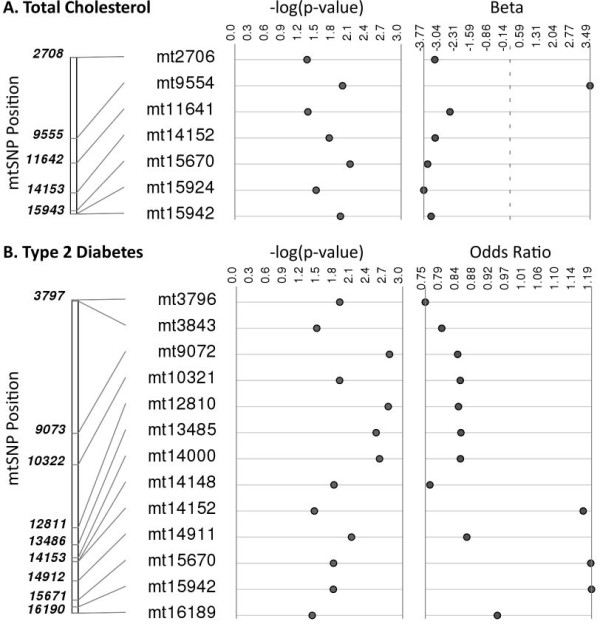
**Mitochondrial SNPs significantly associated with total cholesterol and type 2 diabetes.** Regression analyses were performed to identify mtSNPs associated with: **(A)** total cholesterol and **(B)** type 2 diabetes (T2D). SNPs reaching the significance threshold of p < 0.05 were plotted using Synthesis-View. The -log10 p-values and effect sizes (beta coefficients for total cholesterol and odds ratios for T2D) are shown, plotted in order of base pair position. The dashed line for the beta coefficient values represents no effect on total cholesterol for a given SNP.

Through polygenic modeling we identified total cholesterol and T2D as key traits and proceeded with an mt-PheWAS to determine if the use of polygenic modeling for phenotype prioritization is robust. Linear and logistic regressions adjusted for age, sex, and the first two nuclear PCs were performed for continuous and binary traits, respectively, to investigate the relationship between single mtDNA variants and all phenotypes in this study (Additional file 2). Assuming an uncorrected significance threshold of p <0.05 and considering the number of mtSNPs tested (86), 4.3 SNPs would be expected to be associated by chance alone. More SNPs were significantly associated than would be expected by chance alone for total cholesterol and T2D (Figure 1). Synthesis-view [24] was used to plot mitochondrial SNPs significantly associated with total cholesterol and T2D (Figure 2). The remaining tested traits were associated with zero to five SNPs (Figure 1). These results support our proposed framework of employing polygenic modeling to prioritize phenotypic traits for further analysis. Total cholesterol and T2D exhibited the strongest signals, based on likelihood ratio tests, as well as the greatest number of associated mtSNPs, confirming the expected correlation between PVE and the number of significantly associated mtSNPs.

## Discussion

The goal of this work was to perform an exploratory analysis to establish a framework for mt-PheWAS for investigating the relationship between mtDNA variation and a range of phenotypes. We first employed a polygenic approach to investigate the global effect of mtDNA variation on phenotypic variance for eight cardiovascular-related traits. Given the metabolic trait focus of the nuclear SNP content on the Metabochip and the nature of the selected phenotypes in this study, we expected the polygenic analysis would reveal significant proportion of trait variance explained. However, overall, we observed relatively low PVE for both nuclear and mitochondrial genetic variation. Only a single trait, total cholesterol, reached statistical significance in the polygenic analysis, although T2D approached the statistical significance threshold of p < 0.05. The low PVE may be due, in part, to the targeted nature of the Metabochip which does not contain a genome-wide distribution of SNPs that can be found on GWAS genotyping arrays.

We also performed single mtSNP association analysis to identify mitochondrial genotype-phenotype associations, and to relate the number of significant mtSNPs to the observed mitochondrial PVE for all phenotypes. We found that total cholesterol and T2D, which exhibited the most evidence for a contribution of mitochondrial genetic variation to trait variance or risk, had the highest number of significantly associated mtSNPs. Interestingly, there was some evidence for pleiotropy based on overlap of significantly associated mtSNPs between traits; for example, three mtSNPs (mt14152, mt15670, and mt15942) were significantly associated with both total cholesterol and T2D (Additional file 2).

Studies report inconsistent results on the role of mitochondrial genetic variation in T2D. In the present study we identified 13 mtDNA SNPs associated with T2D in African Americans including variant mt16189, which was previously shown to be correlated with fasting insulin concentration [25], fasting glucose, and BMI [26], and has been associated with T2D in both Asian [27] and European-descent populations [28]. To our knowledge, this is the first reported association of mt16189 with T2D in an African-descent population.

Notably, five of the mtSNPs (mt9072, mt12810, mt13845, mt14000, and mt14911) associated with T2D in our population are common to the African mitochondrial haplogroup L1c [29]. This haplogroup has previously been associated with peripheral neuropathy in HIV patients receiving anti-retroviral therapy in two separate study populations, although with opposite directions of effect [30,31]. Holzinger et al. [31] report a decreased risk of peripheral neuropathy in individuals with the L1c haplogroup. Peripheral neuropathy is a common comorbidity in patients with diabetes and is generally linked to poor control of blood sugar [32]. Our results indicate that mitochondrial haplogroup L1c is associated with decreased risk of T2D. Taken together these data suggest that mitochondrial DNA variation plays a role in both T2D and peripheral neuropathy.

Three mtSNPs (mt14152, mt15670, mt15942) significantly associated with increased risk of T2D in our population are found on the L3e1 African mitochondrial haplogroup which has previously been associated with hypertriglyceridemia in black South Africans on anti-retroviral therapy [33]. Given that high triglyceride levels are associated with increased risk for T2D, this is consistent with our finding that mtSNPs from the L3e1 haplogroup background are associated with increased risk of T2D. We also identified associations between these three SNPs and total cholesterol; however they were negatively correlated with total cholesterol.

While we were successful in establishing a mt-PheWAS framework for future EAGLE and PAGE I studies, our framework is not without limitations. First, the PheWAS approach, even when applied to large datasets such as EAGLE BioVU, can be limited in sample size and thus potentially power depending on the outcomes included in the study. This mt-PheWAS is no exception as the sample sizes available for analysis varied from 1,338 cases of T2D to 9,559 subjects for MCH. Additional limitations of the PheWAS approach include the high-throughput manner in which phenotypes are defined, and that regression models are only minimally adjusted for standard covariates. Because the mt-PheWAS analyses presented herein were exploratory in nature we did not correct for multiple testing. Further work will be necessary for external replication and validation of these results. Finally, as previously mentioned, this present study is limited to genotypic data from the Illumina Metabochip, a custom array designed for fine-mapping specific GWAS-identified regions previously associated with specific phenotypes identified from mostly European-descent populations. The mitochondrial variants included on the Metabochip are also limited, impacting our ability to determine haplogroups for the samples in this study.

## Conclusions

We outlined and tested a framework for performing mt-PheWAS to investigate the relationship between mitochondrial genetic variation and a range of phenotypes. We characterized the utility of polygenic modeling as a method for prioritizing phenotypes for PheWAS by performing both polygenic modeling and comprehensive single mtSNP association testing for a group of eight metabolic traits. Further, we identified multiple single mtSNP associations for total cholesterol and T2D. Our data indicate that, despite relatively limited mtSNP coverage, the Illumina Metabochip is useful in identifying mitochondrial genotype-phenotype associations. Our results also demonstrate that the EAGLE mt-PheWAS framework is capable of identifying known genetic associations and has the potential to uncover novel associations contributing to the complex relationship between human health and mitochondrial genetic variation.

## Abbreviations

BMI: Body mass index; DNA: Deoxyribonucleic acid; EAGLE: Epidemiologic architecture for genes linked to environment; GCTA: Genome-wide complex trait analysis; GRM: Genetic relationship matrix; GWAS: Genome-wide association study; HDL-C: High-density lipoprotein cholesterol; LDL-C: Low-density lipoprotein cholesterol; LRT: Likelihood ratio test; MCH: Mean corpuscular hemoglobin; mtDNA: Mitochondrial deoxyribonucleic acid; mt-PheWAS: Mitochondrial phenome-wide association study; mtSNP: Mitochondrial single nucleotide polymorphism; PAGE: Population architecture using genomics and epidemiology; PCs: Principal components; PheWAS: Phenome-wide association study; PVE: Proportion of variance explained; SD: Standard deviation; SNP: Single nucleotide polymorphism; T2D: Type 2 diabetes.

## Competing interests

The authors have no competing interests to declare.

## Authors’ contributions

SLM was involved in study conception, design, and coordination, as well as drafting and revising the manuscript. JBH conducted the polygenic analyses and was involved in drafting and revising the manuscript. RJG extracted phenotype data from the synthetic derivative and ran single SNP association analyses. JB and EFE extracted phenotype data from the synthetic derivative. SAP was involved in study design and manuscript revision. WSB assisted in extracting phenotype data from the synthetic derivative and manuscript revision. DC was involved in study conception and design, and manuscript revision. All authors read and approved the final manuscript.

## Supplementary Material

Additional file 1 **Mitochondrial and nuclear PVE and LRT p-values.** This table shows data from GCTA, including PVE and SE, for all eight metabolic traits. Click here for file

Additional file 2 **SNP ID, SNP Location, Regression p-values, and number of significant SNPs.** This table shows p-values from regression analysis using PLINK. Click here for file
